# Supplementary material for: Solid-state 31P NMR reveals the biological organophosphorus compounds as the dominant phosphorus species in Saharan dust aerosols
Source: Commun Earth Environ. 2025 Mar 22;6(1):225. doi: 10.1038/s43247-025-02164-w (PMC11929610; doi:10.1038/s43247-025-02164-w)
Supplement: Supplementary file 2 — Supplementary Information [file 43247_2025_2164_MOESM2_ESM.pdf]

Supplementary information

Solid-state ^{31}P NMR reveals the biological organophosphorus compounds as the dominant phosphorus species in Saharan dust aerosols

Kalliopi Violaki[1* #], Christos Panagiotopoulos[1,2#], Claudia Esther Avalos[3,4], Pierre Rossi[5], Ernest Abboud[1], Maria Kanakidou[6,7,8], Athanasios Nenes[1,7]

[1]*Laboratory of Atmospheric Processes and their Impacts, School of Architecture, Civil & Environmental Engineering, École Polytechnique Fédérale de Lausanne, Lausanne 1015, Switzerland*
[2]*Aix Marseille Univ., Université de Toulon, CNRS, IRD, MIO, Marseille, France*
[3]*Institute of Chemical Sciences and Engineering, NMR platform, EPFL, Rte Cantonale, Lausanne 1015, Switzerland*
[4]*Department of Chemistry, New York University, 100 Washington Square East, 10003, NY, USA*
[5]*Central Environmental Laboratory, School of Architecture, Civil & Environmental Engineering, École Polytechnique Fédérale de Lausanne, Lausanne, 1015, Switzerland*
[6]*Environmental Chemical Processes Laboratory (ECPL), Department of Chemistry, University of Crete, P.O. Box 2208, 70013, Heraklion, Greece*
[7]*Center for the Study of Air Quality and Climate Change (C-STACC), Institute of Chemical Engineering Sciences, Foundation for Research and Technology, Hellas, Patras, Greece*
[8]*Institute of Environmental Physics, University of Bremen, Bremen, Germany*

Corresponding author's e-mail:
kalliopi.violaki@epfl.ch
christos.panagiotopoulos@mio.osupytheas.fr

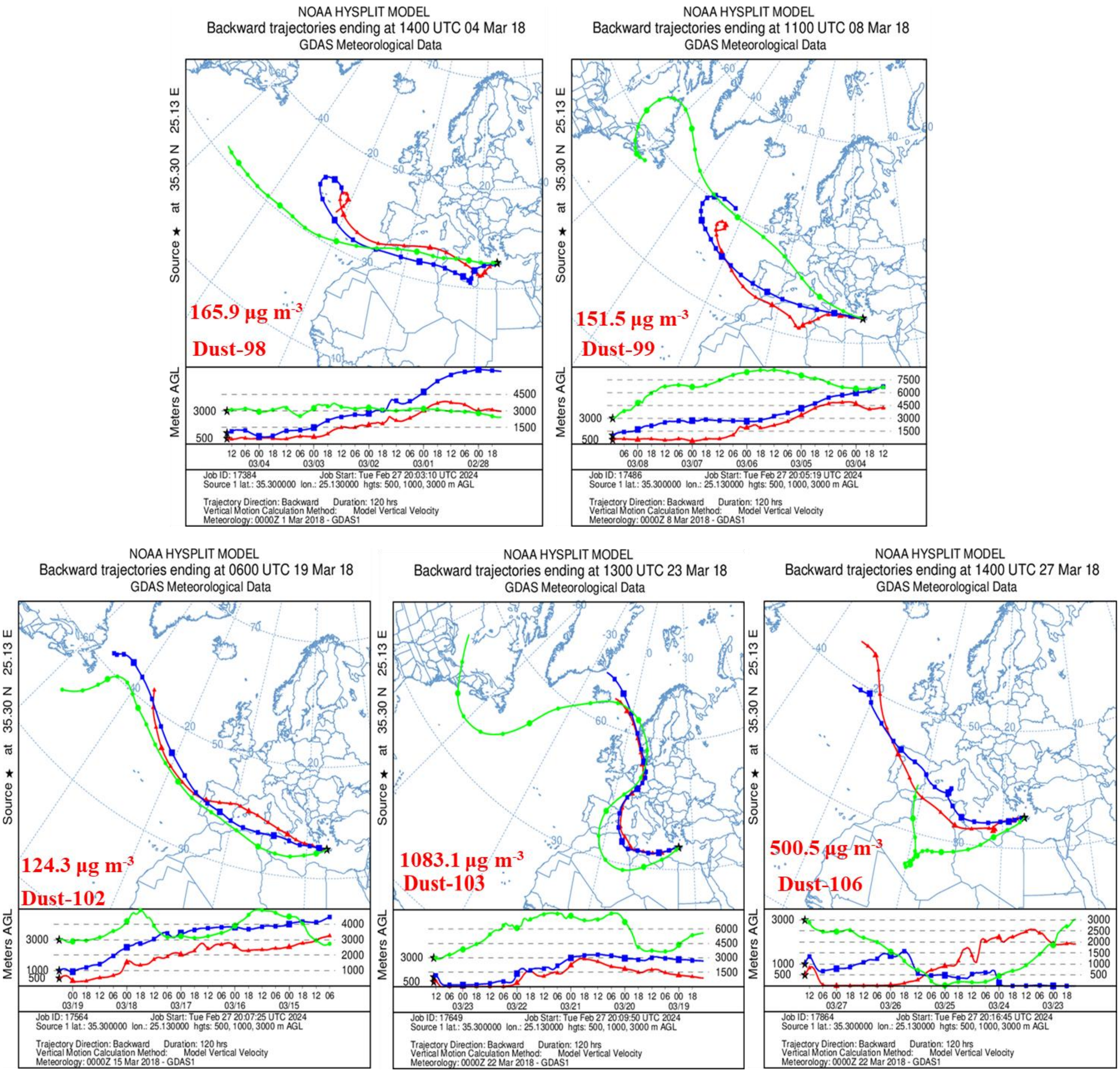

Fig. S1: The five-day back trajectories of the air masses were obtained by using the HYSPLIT model[1] for heights at 500 m, 1000 m and 3000 m. The dust aerosol samples collected from the eastern Mediterranean originated from the NW Sahara Desert. In the left corner is the concentration of TSP atmospheric particulate matter in $\mu g\ m^{-3}$, estimated gravimetrically.

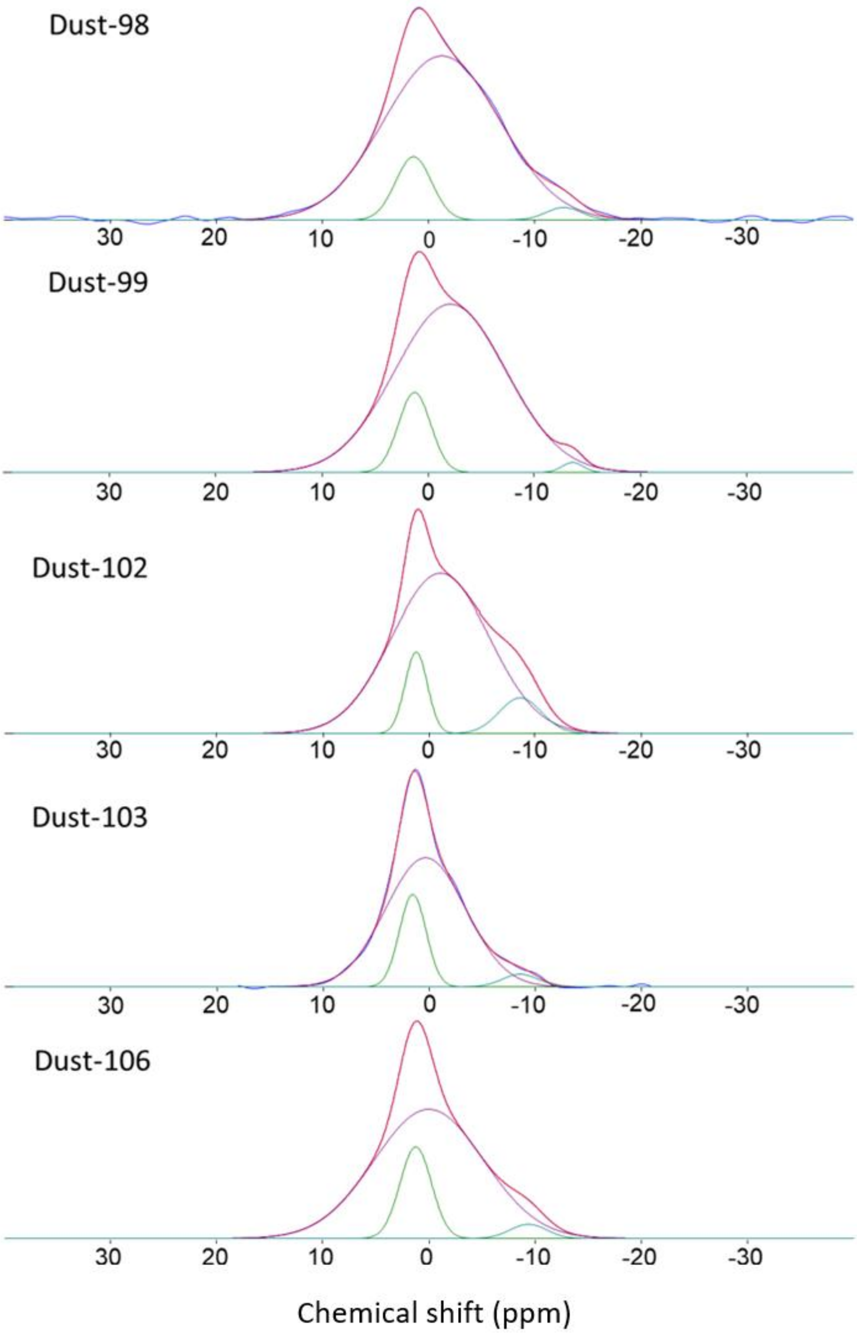

Figure S2. ^{31}P NMR spectra (blue line) and the dmfit Monte Carlo simulation (red line) of all dust samples assessed in this study. Note that the blue line of ^{31}P NMR spectrum of the sample is overlaid by red line of the monte carlo simulation. Fitting parameters were established to include three P-species namely orthophosphate (0-10 ppm; green line), P-diesters (0 to -5 ppm; violet line) and pyrophosphate/polyphosphate (-5 to -20 ppm; dashed line) (See also Fig. 1). Quantification of P-species was made considering the number of scans of the ^{31}P-NMR spectra, the Avogadro number (6.023 x 10^{23}) and the MW of $NH_4H_2PO_4$ used as a reference compound.

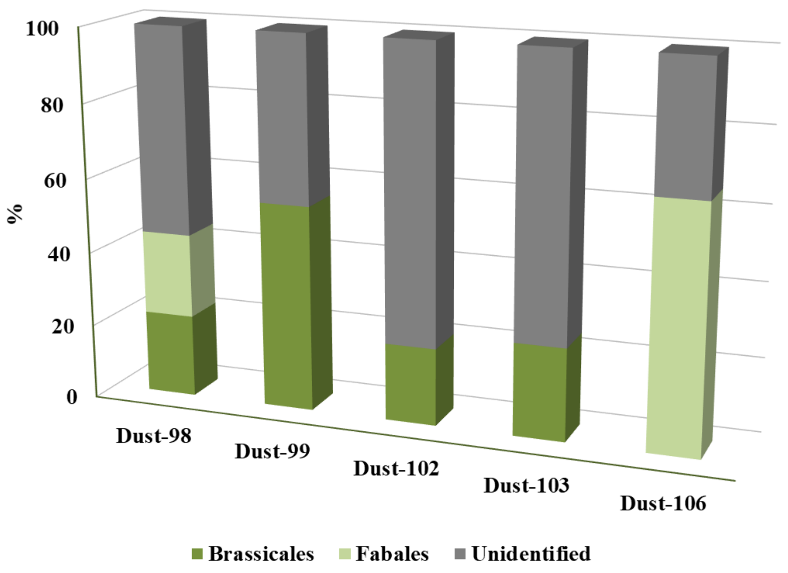

Figure S3. Relative abundance of plants order in the dust samples.

Table S1: Percentage contribution of orthophosphate to TP measured with ion chromatography (IC) and solid-state ^{31}P NMR, respectively. Values for ^{31}P NMR correspond to percentages of peak areas calculated by dividing their areas by the total spectral peak area of the sample (see Fig. 2).

collection date	Sample ID*	Orthophosphate %	
		**^{31}P NMR**	**IC**
02/03/18	Dust-98 (25.1)	10	17
06/03/18	Dust-99 (26.1)	12	16
17/03/18	Dust-102 (6.1)	10	20
21/03/18	Dust-103 (25.7)	19	4
26/03/18	Dust-106 (26.1)	16	10
	Average ± stdev	13 ± 4	13 ± 6

* In parenthesis is the amount of sample in mg disposed for NMR analysis.

Table S2: Pearson correlation coefficient (r) between the main anions, metals, bacterial numbers (as copies of the 16S rDNA gene) per gram of sample, and fungal numbers (as copies of the 18S rRNA gene) gene per gram of sample with P-diesters analyzed in the Saharan dust aerosols (*n=4)*, collected over the eastern Mediterranean area. Only correlations with p <0.05, marked with red, are discussed. XLSTAT statistical software was used (https://www.xlstat.com/en). The dust event on the 21st of March 2018 (Dust-103) was excluded from the statistical analysis as it was the most intense dust episode ever recorded in the area during the 14 years (2004–2018)[2].

Variables	P-diesters	Pyro-P	P-PO4	Bacteria	Fungi	PLs
P-diesters	1					
Pyro-P	0.02	1				
P-PO4	-0.32	-0.56	1			
Bacteria	0.87	-0.03	0.13	1		
Fungi	-0.19	-0.65	0.00	-0.43	1	
PLs	0.92	-0.01	-0.01	0.96	-0.25	1
Sr	0.91	0.15	-0.07	0.96	-0.41	0.98
K	0.92	0.23	-0.16	0.95	-0.48	0.96
Mn	0.92	0.17	-0.11	0.96	-0.42	0.98
Sn	0.05	-0.29	-0.41	-0.32	0.88	-0.07
Sb	0.06	-0.04	-0.76	-0.43	0.51	-0.31
Cs	0.93	0.25	-0.19	0.94	-0.46	0.96
U	0.92	0.13	-0.09	0.96	-0.37	0.99
Fe	0.87	0.08	0.05	0.98	-0.42	0.98
V	0.90	0.18	-0.07	0.97	-0.48	0.97
Cr	0.94	0.18	-0.19	0.93	-0.35	0.98
Cd	-0.01	-0.49	-0.24	-0.35	0.96	-0.12
Pb	0.97	0.08	-0.17	0.94	-0.29	0.98
Co	0.92	0.18	-0.10	0.96	-0.45	0.97
Ni	0.92	0.18	-0.13	0.95	-0.41	0.98
Cu	-0.27	0.89	-0.43	-0.30	-0.35	-0.21
Zn	0.89	-0.08	-0.04	0.88	-0.06	0.98
As	0.88	0.09	0.04	0.99	-0.44	0.98
CL-	0.79	0.07	0.13	0.94	-0.34	0.97
Nitrate	0.24	-0.74	0.07	0.04	0.88	0.22
Sulphate	0.85	-0.10	0.14	0.95	-0.20	0.99
Oxalate	0.88	-0.10	0.14	0.99	-0.29	0.98
Na+	0.80	-0.10	0.14	0.90	-0.12	0.97
NH4+	-0.24	-0.72	-0.02	-0.50	0.94	-0.39
Mg2+	0.84	-0.04	0.14	0.96	-0.27	0.99
Ca2+	0.85	0.01	0.13	0.99	-0.39	0.98

Table S3: Meteorological parameters during the dust events.

Collection date	Sample ID	T (°C)	R (%)	Rain (mm)	Wind Speed (m/sec)
**02-04/03/18**	**Dust-98**	17.9±2.9	61.7±11.2	0.0	12.3±11.3
**06-08/03/18**	**Dust-99**	18.0±3.0	57.6±18.5	0.0	17.8±13.1
**17-19/03/18**	**Dust-102**	17.9±3.1	60.2±19.7	0.0	26.7±18.1
**21-23/03/18**	**Dust-103**	20.8±2.1	39.0±4.8	0.0	32.7±8.5
**26-27/03/18**	**Dust-106**	15.3±2.5	16.0±8.5	0.0	9.5±9.3

S1. Methods

S1.1. DNA extraction, qPCR analysis, NGS sequencing and phylogenetic analysis

DNA extraction: On receipt, the frozen filter samples were handled in a laminar flow hood to avoid contamination. Positive (ZymoBIOMICS Microbial Community Standard, Zymo) and several negative control samples were added to ensure smooth operation and the absence of any form of external contamination throughout the extraction process and subsequent molecular analysis. DNA extraction was carried out in triplicate using the DNeasy PowerSoil Pro kit on a QIAcube Connect robot (all Qiagen) according to the manufacturer's instructions. Prior to extraction, extraction tubes containing extraction buffer and samples were heated to 60°C for 5 minutes, followed by bead beating on a Precellys bead beating system (Bertin Instruments) (2×15 s, at 5500 rpm, with a 20 s interval). 600 µL of the extraction solution was recovered by centrifugation (1 min, 12,000 ×g) and loaded into the QiaCube device. Finally, the DNA was eluted in 100 µL of the final C6 resuspension buffer. Quantification of extracted DNA samples was assessed using fluorometric assays (Qubit ver. 2.0, Life Technologies).

Quantitative PCR analysis: Quantification of the Bacteria 16S rRNA genes was performed in triplicate using the primers 338f (5′-ACTCCTACGGGAGGCAGCAG-3′) and 520r (5′-ATTACCGCGGCTGCTGG-3′). Fungal 18S rRNA was assessed with primers FungiQuant (5′-GGRAAACTCACCAGGTCCAG-3’ and 5′-GSWCTATCCCCAKCACGA-3’) [Liu et al., 2012]. For both analysis, 10 µL reactions were carried out in a MIC qPCR Cycler (BioMolecular Systems, Australia), as follows: 2.5 mL template DNA, 2.1 µL water, 0.2 µL of each primer (10 µM stock) and 5 µL of 2× SensiFAST SYBR® No-ROX Kit (Meridian Bioscience). Samples were cycled (40 cycles) at 95°C for 5 s, followed by extension at 62°C for 15 s and acquisition at 72°C for 15 s. The final melting step was performed from 72°C to 95°C, at a rate of 0.1°C/s. Analysis of the results was performed using the integrated analytical software (micPCR, BioMolecular Systems). On average, efficiency (0.88 – 101.2%) and r2 values (> 0.995) were determined from eight points of serial dilutions (10E8–10E1 copies) of the target gene. Based on the calibration curves, Cq values were used to calculate the gene copy numbers, which were normalized to the mass (ng) of extracted DNA.

Illumina library preparation for bacterial 16S rRNA and sequencing: The hypervariable regions V1–V2 were amplified in a T3000 Thermocycler (Biometra) using 27F and 338R primers with overhang adapters (Table S4). Amplification products were quantified on a

Fragment Analyzer System with a NGS fragment kit (both Agilent) prior to sequencing at the Lausanne Genomic Technologies Facility (University of Lausanne, Switzerland). Multiplex paired-end sequencing (2× 250 bp) was carried out on an Illumina MiSeq platform according to the manufacturer's instructions.

Data were analyzed as shown in Coral et al (2018). Briefly, raw sequencing reads were de-multiplexed and converted to FASTQ files, which were processed on Mothur[3], including denoising with the Single Linkage Preclustering (SLP) method[4] and testing for absence of chimera using the UCHIME algorithm[5] and the SILVA database[6]. The freeware R version 4.0.2 (R Development Core Team, 2020) running on R Studio (version 1.3.1093) was used for inference statistics (alpha diversity and evenness), Mantel tests and numerical ecology analysis. Clustering and Principal Component Analysis (PCA) were carried out on the Hellinger-transformed microbial data set using the Vegan package[7]. Heatmaps were generated using Spearman pairwise correlations between environmental and microbial data sets. Detailed microbial community analysis was performed at the Phylum, Family and Genus taxonomic levels.

Table S4: Bacteria, fungi, and plant primer for the specific region amplification with overhang for Illumina sequencing for bacteria and Oxford Nanopore Sequencing for fungi and plant.

Region	Targeted organisms	Primer name	Sequence with overhang	Reference
16S V1-V2	Bacteria	27F	5'-TCGTCGGCAGCGTCAGATGTGTATAAGAGACAGAGMGTTYGATYMTGGCTCAG-3'	**8**
16S V1-V2	Bacteria	338R	5'-GTCTCGTGGGCTCGGAGATGTGTATAAGAGACAGGCTGCCTCCCGTAGGAGT-3'	**9**
ITS	Fungi	ITS-u4	5'-ACTTGCCTGTCGCTCTATCTTCRGTTTCTTTTCCTCCGCTTA-3'	**10**
18S and ITS	Plant	Fw1	5'-TTTCTGTTGGTGCTGATATTGCTAGAGGAASTAAAAGTCGTAA-3'	**11**
18S and ITS	Plant	RGN3	5'-ACTTGCCTGTCGCTCTATCTTCTCCTGAGGGAAACTTCG-3'	**11**

Oxford Nanopore library preparation for Fungi and pollen ITS and sequencing: For each sample, 20 µL of pollen and Fungi amplicons obtained following the manufacturer conditions were pooled and purified using AMPure XP magnetic beads (Beckman Coulter). Quantification was assessed using fluorometric assays (Qubit ver. 2.0, Life Technologies) and 100-200 fmol of each sample was transferred to a 0.2 ml clear PCR tube for barcoding according to ONT instructions. All barcoded samples were pooled and purified with AMPure XP magnetic beads (Beckman Coulter) prior to End-prep reaction (Ultra II End-prep Enzyme Mix, NEB) and adapter ligation (NEBNext Quick T4 DNA Ligase, NEB) following the manufacturers' instructions.

Sequencing was carried out on a MinION sequencer (ONT) using a R9.4.1 FLO-MIN 106 flow cells for up to 48 hours. Primary acquisition, real-time basecalling and demultiplexing were carried out using the graphical user interface MinKNOW v2.0 and Guppy basecaller v3.0.6 for fungi and with Dorado v0.8.3.

After demultiplexing, FASTQ files were imported into the public server usegalaxy.eu for further analysis. For pollen, Nanopore adapters and barcode were first trimmed using the Dorado v0.8.3 command line and then imported to Galaxy. For fungi, Nanopore adapters and chimeric sequences were trimmed using the Porechop tool from the NanoGalaxy toolbox[12]. Subsequently, sequences were filtered with the Fastp tool[10], applying a quality phred score of 913. Data was then partitioned into two sub-datasets with lengths ranging between 700-1500 bp and 1400–1800 bp (for pollen and fungi, respectively) and overall quality was assessed using FastQC and MultiQC. Taxonomic classification was performed using the Kraken2 tool with a confidence threshold of 0.1, utilizing database[14] SILVA 2022. Taxonomic assignments were visualized using Krona pie charts[15] and data was imported into RStudio using the Pavian application[16]. Detailed taxonomical analysis were performed at the Phylum, Class, Order, and Genus levels.

Supplementary References

1. Stein, A.F., Draxler, R.R, Rolph, G.D., Stunder, B.J.B., Cohen, M.D., and Ngan, F., (2015). NOAA's HYSPLIT atmospheric transport and dispersion modeling system, Bull. Amer. Meteor. Soc., 96, 2059-2077, http://dx.doi.org/10.1175/BAMS-D-14-00110.
2. Solomos, S.; Kalivitis, N.; Mihalopoulos, N.; Amiridis, V.; Kouvarakis, G.; Gkikas, A.; Binietoglou, I.; Tsekeri, A.; Kazadzis, S.; Kottas, M.; et al. From Tropospheric Folding to Khamsin and Foehn Winds: How Atmospheric Dynamics Advanced a Record-Breaking Dust Episode in Crete. *Atmosphere* **2018**, *9*, 240.
3. Schloss PD et al. 2009. Introducing mothur: Open-source, platform-independent, community-supported software for describing and comparing microbial communities. Applied and Environmental Microbiology 75:7537–7541.
4. Huse, S. M., Welch, D. M., Morrison, H. G., & Sogin, M. L. (2010). Ironing out the wrinkles in the rare biosphere through improved OTU clustering. Environmental microbiology, 12(7), 1889-1898.
5. Edgar, R. C., Haas, B. J., Clemente, J. C., Quince, C., & Knight, R. (2011). UCHIME improves sensitivity and speed of chimera detection. Bioinformatics, 27(16), 2194-2200.
6. Glöckner, F. O., Yilmaz, P., Quast, C., Gerken, J., Beccati, A., Ciuprina, A., & Ludwig, W. (2017). 25 years of serving the community with ribosomal RNA gene reference databases and tools. Journal of biotechnology, 261, 169-176.
7. Oksanen, J., Blanchet, F.G., Kindt, R., Legendre, P., Minchin, P.R., O'Hara, R.B., Simpson, G.L., Solymos, P., Stevens, M.H.H. and Wagner, H. (2014) Vegan: Community Ecology Package. R Package Version 2.2-0. http://CRAN.Rproject.org/package=vegan
8. Wilson, K. H., Blitchington, R. B., & Greene, R. C. (1990). Amplification of bacterial 16S ribosomal DNA with polymerase chain reaction. Journal of clinical microbiology, 28(9), 1942-1946.

9. Muyzer, G., Teske, A., Wirsen, C. O., & Jannasch, H. W. (1995). Phylogenetic relationships of Thiomicrospira species and their identification in deep-sea hydrothermal vent samples by denaturing gradient gel electrophoresis of 16S rDNA fragments. Archives of Microbiology, 164, 165-172.
10. Cheng T, Xu C, Lei L, Li C, Zhang Y, Zhou S. Barcoding the kingdom Plantae: new PCR primers for ITS regions of plants with improved universality and specificity. (2016). Mol Ecol Resour. 16(1):138-49.
11. Ohta, A., Nishi, K., Hirota, K. *et al.* (2023). Using nanopore sequencing to identify fungi from clinical samples with high phylogenetic resolution. *Sci Rep* **13**, 9785
12. Wick, R. (2017). Porechop. In GitHub repository. GitHub. https://github.com/rrwick/Porechop
13. Nygaard et al., 2020: A preliminary study on the potential of Nanopore MinION and Illumina MiSeq 16S rRNA gene sequencing to characterize building-dust microbiomes
14. Wood, D. E., & Salzberg, S. L. (2014). Kraken: ultrafast metagenomic sequence classification using exact alignments. Genome Biology, 15(3), R46. https://doi.org/10.1186/gb-2014-15-3-r46
15. Ondov, B. D., Bergman, N. H., & Phillippy, A. M. (2011). Interactive metagenomic visualization in a Web browser. BMC Bioinformatics, 12(1). https://doi.org/10.1186/1471-2105-12-385
16. Breitwieser FP, Salzberg SL. Pavian: interactive analysis of metagenomics data for microbiome studies and pathogen identification. Bioinformatics. 2020 Feb 15;36(4):1303-1304. doi: 10.1093/bioinformatics/btz715. PMID: 31553437; PMCID: PMC8215911.
