# Supplementary material for: Solid-state 31P NMR reveals the biological organophosphorus compounds as the dominant phosphorus species in Saharan dust aerosols
Source: Commun Earth Environ. 2025 Mar 22;6(1):225. doi: 10.1038/s43247-025-02164-w (PMC11929610; doi:10.1038/s43247-025-02164-w)
Supplement: Supplementary file 3 — Reporting Summary [file 43247_2025_2164_MOESM3_ESM.pdf]

nature portfolio

Corresponding author(s): COMMSENV-24-2776B

Last updated by author(s): Feb 14, 2025

Reporting Summary

Nature Portfolio wishes to improve the reproducibility of the work that we publish. This form provides structure for consistency and transparency in reporting. For further information on Nature Portfolio policies, see our Editorial Policies and the Editorial Policy Checklist.

Statistics

For all statistical analyses, confirm that the following items are present in the figure legend, table legend, main text, or Methods section.

n/a	Confirmed	
☐	☒	The exact sample size (*n*) for each experimental group/condition, given as a discrete number and unit of measurement
☐	☒	A statement on whether measurements were taken from distinct samples or whether the same sample was measured repeatedly
☒	☐	The statistical test(s) used AND whether they are one- or two-sided<br>*Only common tests should be described solely by name; describe more complex techniques in the Methods section.*
☐	☒	A description of all covariates tested
☐	☒	A description of any assumptions or corrections, such as tests of normality and adjustment for multiple comparisons
☐	☒	A full description of the statistical parameters including central tendency (e.g. means) or other basic estimates (e.g. regression coefficient) AND variation (e.g. standard deviation) or associated estimates of uncertainty (e.g. confidence intervals)
☒	☐	For null hypothesis testing, the test statistic (e.g. *F*, *t*, *r*) with confidence intervals, effect sizes, degrees of freedom and *P* value noted<br>*Give P values as exact values whenever suitable.*
☒	☐	For Bayesian analysis, information on the choice of priors and Markov chain Monte Carlo settings
☐	☒	For hierarchical and complex designs, identification of the appropriate level for tests and full reporting of outcomes
☐	☒	Estimates of effect sizes (e.g. Cohen's *d*, Pearson's *r*), indicating how they were calculated

Our web collection on statistics for biologists contains articles on many of the points above.

Software and code

Policy information about availability of computer code

Data collection	*Provide a description of all commercial, open source and custom code used to collect the data in this study, specifying the version used OR state that no software was used.*
Data analysis	Data analysis from NMR were performed using the dmfit program (https://nmr.cemhti.cnrs-orleans.fr/dmfit/default.aspx).

For manuscripts utilizing custom algorithms or software that are central to the research but not yet described in published literature, software must be made available to editors and reviewers. We strongly encourage code deposition in a community repository (e.g. GitHub). See the Nature Portfolio guidelines for submitting code & software for further information.

Data

Policy information about availability of data

All manuscripts must include a data availability statement. This statement should provide the following information, where applicable:

- Accession codes, unique identifiers, or web links for publicly available datasets
- A description of any restrictions on data availability
- For clinical datasets or third party data, please ensure that the statement adheres to our policy

Provide your data availability statement here.

Research involving human participants, their data, or biological material

Policy information about studies with human participants or human data. See also policy information about sex, gender (identity/presentation), and sexual orientation and race, ethnicity and racism.

Reporting on sex and gender	This information is not relevant with this study.
Reporting on race, ethnicity, or other socially relevant groupings	This information is not relevant with this study.
Population characteristics	This information is not relevant with this study.
Recruitment	This information is not relevant with this study.
Ethics oversight	This information is not relevant with this study.

Note that full information on the approval of the study protocol must also be provided in the manuscript.

Field-specific reporting

Please select the one below that is the best fit for your research. If you are not sure, read the appropriate sections before making your selection.

☐ Life sciences ☐ Behavioural & social sciences ☒ Ecological, evolutionary & environmental sciences

For a reference copy of the document with all sections, see nature.com/documents/nr-reporting-summary-flat.pdf

Ecological, evolutionary & environmental sciences study design

All studies must disclose on these points even when the disclosure is negative.

Study description	Saharan dust phosphorous is dominated by organic biological sources, accounting for 83% of total phosphorous, according to the analysis of dust particle samples performed with solid-state 31P NMR technique.
Research sample	The research samples (n=5) are atmospheric particles (aerosols) from Saharan dust.
Sampling strategy	The analyzed samples were chosen based on the collected mass due to the limitation of NMR technique. During the dust events usually the particulate mass on the filter is higher that 15 mg.
Data collection	K.V., C.P. and C.E.A. collected and analyzed the data by running the samples in NMR, Ion chromatography, LC-MS-TOF, ICP-MS. P.R and E.A. produced the microbiology data.
Timing and spatial scale	The dust samples collected only during the a Saharan dust event was taking place in Crete during March 2018.<br>The collection dates were:<br>1. 02-04/03/18 2. 06-08/03/18 3. 17-19/03/18 4. 21-23/03/18 5. 26-27/03/18
Data exclusions	No data were excluded from the analysis.
Reproducibility	All attempts to analyze the data were successful.
Randomization	This is an interdisciplinary study in which chemists and biologists collaborate to produce high-impact results. The scientists were based at the EPFL campus.
Blinding	Blinding was not relevant in this study because the purpose of this study is to investigate the impact of atmospheric P from the Saharan dust in biogeochemical P cycle.

Did the study involve field work? ☒ Yes ☐ No

Field work, collection and transport

Field conditions	The meteorological condition during the collection of the samples were described in Supplementary Table S3.
Location	The samples (n=5) were collected in March 2018 from Crete, Greece.
Access & import/export	Prof. Kanakidou (co-author in the paper) was responsible for the technical part of the field campaign.

Disturbance	No disturbance

Reporting for specific materials, systems and methods

We require information from authors about some types of materials, experimental systems and methods used in many studies. Here, indicate whether each material, system or method listed is relevant to your study. If you are not sure if a list item applies to your research, read the appropriate section before selecting a response.

Materials & experimental systems

n/a	Involved in the study
☒	☐ Antibodies
☒	☐ Eukaryotic cell lines
☒	☐ Palaeontology and archaeology
☒	☐ Animals and other organisms
☒	☐ Clinical data
☒	☐ Dual use research of concern
☒	☐ Plants

Methods

n/a	Involved in the study
☒	☐ ChIP-seq
☒	☐ Flow cytometry
☒	☐ MRI-based neuroimaging

Plants

Seed stocks	This information is not relevant with this study.
Novel plant genotypes	This information is not relevant with this study.
Authentication	This information is not relevant with this study.
